# Advancing Pharmaceutical Care in Community Pharmacies in Poland: A Blueprint for Enhanced Patient Care Quality

**DOI:** 10.3390/healthcare12212109

**Published:** 2024-10-23

**Authors:** Piotr Merks, Anna Kowalczuk, Aleksandra Howell, Artur Białoszewski, Justyna Strocka, Ewa Krajewska, Jarosław Pinkas, Janusz Ostrowski, Anna Staniszewska, Agnieszka Neumann-Podczaska, Melania Brzozowska, Anna Augustynowicz, Mariola Borowska, Agnieszka Drab, Jolanta Herda, Justyna Kaźmierczak, Urszula Religioni

**Affiliations:** 1 Faculty of Medicine, Collegium Medicum, Cardinal Stefan Wyszyński University, Wóycickiego 1/3, 01-938 Warsaw, Poland; 2 Agency for Health Technology Assessment and Tariff System, 00-032 Warsaw, Poland; 3 Nuffield Department of Medicine, University of Oxford, Oxford OX3 7BN, UK; 4 Department of the Prevention of Environmental Hazards, Allergology and Immunology, Medical University of Warsaw, 02-097 Warsaw, Poland; artur.bialoszewski@wum.edu.pl; 5 School of Public Health, Centre of Postgraduate Medical Education, 01-813 Warsaw, Polandjaroslaw.pinkas@cmkp.edu.pl (J.P.);; 6 Main Pharmaceutical Inspectorate, 00-082 Warsaw, Poland; 7 Department of Experimental and Clinical Pharmacology, Medical University of Warsaw, 02-091 Warsaw, Poland; 8 Department of Palliative Medicine, Poznan University of Medical Sciences, 61-245 Poznan, Poland; 9 Drug Reimbursement Department, National Health Fund, 02-528 Warsaw, Poland; 10 Department of Health Economics and Medical Law, Medical University of Warsaw, 01-445 Warsaw, Poland; 11 Department of Cancer Epidemiology and Primary Prevention, Maria Sklodowska-Curie National Research Institute of Oncology, 02-781 Warsaw, Poland; mariola.borowska@nio.gov.pl; 12 Department of Medical Informatics and Statistics with e-Health Lab, Medical University of Lublin, 20-124 Lublin, Poland; 13 Department of Public Health, Medical University of Lublin, 20-124 Lublin, Poland; 14 The Polish Pharmacy Practice Research Network (PPPRN), 01-938 Warsaw, Poland

**Keywords:** pharmaceutical care, community pharmacy, quality of life, pharmacy service

## Abstract

**Background**: This article reviews the current state of pharmaceutical care in community pharmacies in Poland and proposes a collaborative framework for its advancement. While pharmaceutical care has evolved significantly worldwide, with Europe leading the way, Poland has lagged in its development. Although Polish pharmacists are well-qualified and community pharmacies are numerous, pharmaceutical care remains underdeveloped. **Methods**: We conducted a literature review and analyzed case studies from European countries with advanced pharmaceutical services. Based on these findings, we collaborated with policy makers, commissioners, and academics to develop a framework for enhancing pharmaceutical care in Poland. The plan emphasizes integrating seven key services into Polish community pharmacies. **Results**: Our proposed framework outlines seven essential pharmaceutical services: medicine use reviews, new medicine services, minor ailment services, repeat prescription services, integrated prevention programs, cardiovascular disease prevention programs, and vaccination programs. Evidence from other European countries suggests that implementing these services could significantly improve health outcomes and patient quality of life. This is particularly important in light of Poland’s ageing population, the rising prevalence of chronic diseases, and the healthcare system’s increasing burden due to polypharmacy. **Conclusions**: The proposed framework presents a practical and collaborative approach to advancing pharmaceutical care in Poland. By adopting these key services, community pharmacies could play a more integral role in improving patient care quality and alleviating pressure on the broader healthcare system.

## 1. Introduction

The Pharmaceutical Care Network Europe (PCNE) defines pharmaceutical care as a pharmacist’s contribution to the care of individuals in order to optimize medicine use and improve health outcomes [1]. This entails identifying and solving potential and existing drug-related problems (DRPs), most effectively achieved through close collaboration between patients, pharmacists, and other healthcare professionals, predominantly doctors. To address DRPs, comprehensive pharmaceutical care plans integrated into overall patient treatment schemes are essential. Key elements of pharmaceutical care encompass patient counselling on medications and supporting healthcare systems, particularly in the context of prescribing [2].

However, an important actor in this relationship that is often overlooked is the pharmaceutical industry. Pharmaceutical companies play a key role in the development and distribution of medicines, which influences pharmacists’ ability to provide effective care and address DRPs. Their collaboration is essential in ensuring that patients have access to appropriate medications.

The World Health Organisation (WHO) underscores the importance of integrating pharmacists into healthcare teams, emphasizing close collaboration between pharmacists and other healthcare professionals to maximize health outcomes [3,4,5]. In Poland, medicines—both prescription and over-the-counter (OTC)—are sold primarily in pharmacies, with pharmacists acting as gatekeepers. Even OTC drugs require pharmacist oversight, a unique feature of the Polish healthcare system, unlike in many other countries where OTC medications are more freely available without professional involvement.

The European Council, in 2000, highlighted the benefits of pharmaceutical care for patients, recommending its incorporation into national healthcare systems [6]. In recent years, several countries have developed advanced pharmaceutical care plans [7]. Despite the explicit definition of pharmaceutical care in the Pharmaceutical Chambers Act in Poland [8], involving pharmacist collaboration with patients, doctors, and, if necessary, other healthcare professionals, the actual implementation has been notably low. Recognizing this gap, recent initiatives have been planned in Poland to elevate the level of pharmaceutical care and align it with the defined objectives.

## 2. The Role of Pharmaceutical Care in Healthcare Systems

Community pharmacies, being the most frequently visited healthcare facilities, present a prime opportunity for a diverse array of activities aimed at maximising health outcomes [9,10]. Pharmacists can play a crucial role by offering various services, including designing and implementing therapeutic plans; identifying, explaining, and resolving drug-related problems; organising educational campaigns; and facilitating laboratory and screening tests [11].

These activities are of particular importance in the context of our ageing society, which is witnessing a rise in individuals with chronic diseases and cancers [12,13]. This demographic shift leads to potentially inappropriate polypharmacy, i.e., the administration of multiple medications exceeding medical necessity, requiring vigilant monitoring of prescribed products [14,15,16]. Polypharmacy, understood as situations where patients take five or more medications [14], disproportionately affects the elderly, with nearly 50% of those aged over 65 taking unnecessary medications, often leading to problems such as double therapies and contraindicated combinations [17,18].

Self-medication has become more common in Poland during the COVID-19 pandemic, as patients often bypassed healthcare professionals due to limited access to doctors and hospitals [19]. This increase in self-medication has highlighted both the importance of pharmacists as trusted advisors on the safe use of medications and the risks associated with unsupervised use of medications, leading to potential medication-related problems. Trust in pharmacists, doctors, and vaccines has clearly been affected by the pandemic, with mixed results. While pharmacists have gained greater recognition for their role in healthcare, particularly through the administration of vaccines, skepticism about vaccines and medicines has increased in some groups, complicating efforts to improve public health outcomes [20].

Non-adherence to prescribed treatments, affecting approximately 50% of patients, poses another significant challenge [21,22]. The consequences of non-adherence include health deterioration, worsening comorbidities, and, in extreme cases, fatalities [23]. The economic impact is substantial, with an average annual cost per non-adherent patient ranging from USD 949 to 44,190 [21].

Pharmaceutical care serves as an exemplar of integrated services involving diverse medical professions. The WHO emphasizes that pharmaceutical services should be delivered in a manner that ensures patients receive continuous preventive and medical care tailored to their needs across different levels of the healthcare system. Integrated services streamline provision, minimising stages of meetings and separate visits, facilitating effective communication among healthcare staff from different facilities to ensure continuous and high-quality patient care [24].

The implementation of comprehensive pharmaceutical care in healthcare systems promises multiple health benefits, optimising clinical outcomes for patients, alleviating the strain on healthcare systems, and—through proactive identification and resolution of drug-related problems—minimising unnecessary medical visits or hospitalisations, thereby resulting in substantial financial benefits.

## 3. Pharmaceutical Services in Poland and Worldwide

Pharmaceutical services exhibit notable variations among different countries, with recent years witnessing significant evolution [25]. A study by the Institute for Evidence-Based Healthcare highlights that Medicine use reviews are the most prevalent services offered in European pharmacies [26]. Other common services include medication dosing support programmes, medical device usage training, and pharmacist-administered vaccinations, as seen in countries like in Canada, Australia and Great Britain. Some countries offer services related to initial medication dispensing, adherence support, and ongoing medical guidance [26,27]. Additional services may include treating minor ailments and conducting screening tests for early diagnosis (Pharmacy First Service, PFS) [28,29].

In Poland, the sole reimbursement service pertains to the preparation of compound medications. Amendments to the Pharmacist Profession Act will soon permit the continuation of medical instruction by pharmacists. Currently, there are no publicly funded pilot programmes for pharmaceutical care in the country. European nations, on the other hand, implement screening programmes for at-risk patients, covering conditions such as diabetes, hypertension, HIV, hepatitis, colorectal cancer and lipid disorders, along with pharmaceutical consultations for initial medication dispensing [26].

The role of pharmacists in healthcare has been particularly pronounced since the COVID-19 pandemic. With many healthcare facilities closed, pharmacists assumed a crucial role in advising patients on medications, significantly alleviating the strain on primary healthcare. Presently, global efforts are underway to administer COVID-19 vaccinations. A key challenge lies in ensuring widespread access to vaccinations. Countries that have empowered pharmacists to administer other vaccinations, such as the flu vaccine, can leverage this professional group to facilitate COVID-19 vaccinations and enhance accessibility.

## 4. Arguments for Enhancing Pharmaceutical Care in Poland

The aging demographic trend in Poland, as projected by the Central Statistical Office (GUS), foresees a population exceeding 11 million individuals who are aged over 65 by 2050 [30]. This demographic shift could place significant strain on the public budget, with the latest data indicating that 42% of public health expenditures are directed towards patients aged over 60 [31,32]. With a growing elderly population, these expenditures are poised to increase.

Elderly individuals constitute the predominant group reliant on prescription medications, with Eurostat reporting that 13.8% of Polish seniors receive prescription medications (slightly below the EU average). Notably, 51.8% of seniors in Poland use over-the-counter (OTC) medications, surpassing the European average. Moreover, medication consumption increases with age, necessitating specialised treatment monitoring.

The insufficient number of medical staff, particularly doctors and nurses, represents another compelling argument for the introduction of pharmaceutical care in Poland. In 2018, there were 2.4 doctors per 1000 inhabitants, the lowest ratio among all European Union states, with the highest number of consultations per doctor [33].

Pharmaceutical services offer a viable solution to relieve the burden on the healthcare system. Poland boasts a pharmacy and pharmacist density (36 and 77 per hundred thousand inhabitants, respectively) in line with the European average [34]. The relatively low number of individuals visiting a pharmacy or pharmacy outlet (approximately 2700), suggests untapped potential for more comprehensive patient care [35]. Furthermore, studies carried out in Poland indicate the high level of public trust in pharmacists [36].

These considerations collectively indicate that, given the accessibility of pharmacies and public trust, pharmacists could assume a substantial role in various healthcare activities. This includes repeat prescription service, managing minor ailments, monitoring the efficacy and safety of chronic disease therapies, administering vaccinations and other injections, and conducting screening tests.

## 5. Policies and Strategic Plans

The Team for Pharmaceutical Care—consisting of representatives of the Ministry of Health, the National Health Fund, the National Pharmacy Chamber and the academic community—collaboratively produced a document entitled “Report. Pharmaceutical Care. A Comprehensive Analysis of the Implementation Process” [37]. This report lays the foundation for the introduction of pharmaceutical care services into the healthcare system in Poland, offering strategic suggestions that can be implemented in the coming years, subject to the necessary legal requirements. This document is instrumental for the effective integration of pharmaceutical care in Poland. The following are the key assumptions outlined in the report.

### 5.1. Medicine Use Reviews (MURs)

The medicine use review (MUR) is a structured patient interview designed to enhance patient understanding of their medications and optimise pharmacotherapy [38]. This comprehensive review encompasses all medications, including dietary supplements and specific nutritional products. MURs aim to identify potential drug-related problems and interactions while educating patients to improve adherence, leading to enhanced health outcomes [39,40,41,42]. Given the prevalent use of multiple medications, particularly among the elderly, it is recommended that individuals at risk of polypharmacy (simultaneous administration of five or more medications) and those with medical recommendations for reviews (for example, after changing treatments prescribed by doctor) should automatically qualify for MURs.

Several pilot MUR programmes have been conducted in Polish community pharmacies, including OF-Senior [43]. This initiative involved 291 reviews among patients aged ≥ 65 years (mean age 74.9 ± 74.3) with significant polypharmacy of ≥10 pharmaceutical products.

The proposed pilot programme will assess the effectiveness of MURs in community pharmacies and evaluate the service’s utility beyond the programme’s duration. To enable pharmacists’ access to the patients’ list of medications stored in their medical records, the electronic health record MUR module of the Pharmaceutical Care System will be used.

### 5.2. New Medicine Service (NMS)

Non-adherence to new medications affects nearly 30% of patients within the initial days of treatment [44]. To address this, the New Medicine Service (NMS) has been implemented in pharmaceutical practice in multiple countries [45,46]. Its objective is to support pharmacists to assist patients in taking new medications. Studies show that NMS significantly improves adherence, increases medication safety, optimises health outcomes, and enhances patient involvement in their treatment [45,46].

In Poland, the NMS was included in the pilot programme “Closer to the Patient”, aimed at patients diagnosed with atrial fibrillation receiving prescriptions for a new-generation anticoagulant (dabigatran) for the first time [47]. Findings from this study indicate that pharmaceutical interventions in the study group result in significantly improved adherence to pharmacotherapy.

The NMS will comprise three pharmaceutical consultations within the first month of initiating a new medication, with a minimum interval of one week and the option for online consultations. Targeting patients receiving a prescription for a medication used in a chronic disease treatment for the first time, the service aims to educate patients by providing comprehensive information on their medications, offer personalised lifestyle advice, ensure pharmacist supervision over treatment efficacy in the initial weeks, and evaluate treatment tolerance and effectiveness.

### 5.3. Minor Ailment Service (MAS)

The primary goals of implementing the Minor Ailment Service (MAS) within pharmaceutical care are to increase the access to healthcare and alleviate the strain on healthcare facilities, specifically general practitioner (GP) consultations for minor ailments, where pharmacists can provide valuable assistance. MAS has been successfully introduced to community pharmacies in several countries [48,49,50], resulting in improved doctor availability for other patients and cost savings. In Poland, doctors provided advice on 23 million minor ailments in 2019 [51], a service that pharmacists could potentially handle, thereby optimising resource utilisation. To facilitate this, the Team for Pharmaceutical Care has compiled a list of 162 diagnoses (ICD-10 codes) for minor ailments that pharmacists can effectively address. Establishing clear guidelines and conditions for the treatment of these specific conditions is crucial for enabling pharmacist consultations on minor ailments in community pharmacies in Poland.

### 5.4. Repeat Prescription Service

The objective of repeat prescriptions is to ensure the uninterrupted continuation of patient treatment. Enabling pharmacists to issue repeat prescriptions simplifies medication purchases for patients and significantly relieves the burden on healthcare facilities. This is of particular significance as visits related to repeat prescriptions constitute approximately 15% of all healthcare facility visits in Poland [52].

Amidst the COVID-19 pandemic, many countries have empowered pharmacists to issue repeat prescriptions. In Poland, however, pharmacists were initially limited to issuing such prescriptions only in life and health-threatening situations, starting in 2020 [53]. The extensive informatisation of healthcare in Poland and the introduction of e-prescriptions provide an opportunity to swiftly alleviate the burden on primary care physicians. At last, pharmacists in Poland will be able to provide repeat prescription service and issue prescriptions for medicinal product refills, nutritional products, and medicinal devices based on medical orders stored in the Medical Information System (MIS). These orders will remain valid for 12 months from issuing, although the initial recommendation proposed a 6-month validity to ensure regular monitoring of patients with chronic conditions (REF) [54].

### 5.5. Integrated Prevention Programme

Although several preventive screening programs in Poland are publicly funded, the system lacks effective integration and coordination of these initiatives, which limits the ability to compile comprehensive patient health information necessary for ensuring high-quality care. The Integrated Prevention Programme seeks to close this gap by collecting detailed patient health data and engaging at-risk groups in screening tests. This initiative is built around standardized electronic patient surveys tailored to each preventive program, along with a screening qualification questionnaire and the Information System for Prevention Monitoring. A key element of this programme is involving pharmacists in conducting screening and prevention tests. By leveraging their professional role, pharmacists can actively encourage patients to complete the surveys and participate in preventive programmes, such as the cardiovascular disease prevention initiative.

### 5.6. Cardiovascular Disease Prevention Programme

Cardiovascular diseases account for over 40% of all deaths in Poland [55]. Currently, GPs in Poland are responsible for conducting cardiovascular disease prevention programmes. The Pharmacist Profession Act underscores pharmacists’ participation in health education, promoting healthy lifestyles, and preventive medicine. Given that prevention programmes often involve pharmacotherapy, the involvement of pharmacists in conducting these programmes has been proposed. Drawing from successful experiences in other countries [56,57,58], it is recommended that pharmacists issue referrals for diagnostic tests or even perform some tests within pharmacies.

As part of these cardiovascular disease prevention programs, during a pharmaceutical consultation, pharmacists would interview patients and complete the prevention test report. Patients showing indications of cardiovascular disease would be referred to doctors for further investigations. This collaborative approach, integrating pharmacists into prevention initiatives, aims to enhance accessibility, promote early detection, and contribute to a more comprehensive and proactive approach to cardiovascular health within the healthcare system.

### 5.7. Vaccinations in Community Pharmacies

In various countries, community pharmacies have successfully incorporated vaccinations as an important component of public health prevention and protection [59,60,61]. Studies show that the implementation of vaccinations in community pharmacies significantly boosts vaccination coverage levels and eases the burden on healthcare facilities [62]. Vaccinations administered by pharmacists in community pharmacies are of particular importance in the face of the COVID-19 pandemic.

Given the notably low vaccination coverage in Poland (for example, in the case of influenza vaccines, coverage is approximately 7%, compared to 70% in Great Britain) [63], introducing vaccinations in community pharmacies can increase their availability and popularity among patients.

Although current Polish law allows only doctors to qualify patients for vaccinations [64], there is a proposal to extend this authority to nurses and pharmacists through a specialised questionnaire. Pharmacies intending to administer vaccinations would need to meet specific requirements, including technical and staff requirements [65]. Pharmacists administering vaccinations would undergo specialised training. To initiate the pilot programme in pharmacies in Poland, influenza vaccinations will be prioritised, aiming to facilitate access and increase vaccination coverage among patients at risk of post-influenza complications. This initiative seeks to minimise visits to healthcare facilities, while offering greater convenience to patients.

### 5.8. Other Pharmaceutical Care Services

Tailoring pharmaceutical services to the needs of patients in the Polish healthcare system allows for potential expansions. These may include preventative measures against smoking-attributable diseases, provision of syringes and needles, targeted MURs for specific groups of medications (e.g., non-steroidal anti-inflammatory drugs, antiplatelet medications, diuretics, medications with a narrow therapeutic index), and support for patients with long-term conditions. The latter suggestion was piloted and evaluated in the Pharmaceutical Care in Hypertension (PCH) programme and its continuation, the Pharmaceutical Care in Hypertension and Diabetes (PCHD) programme [66,67,68]. These long-term intervention studies revealed that pharmaceutical care, compared to standard care, improved patient knowledge of their disease and positively impacted hypertension management. Notably, in the PCHD study, pharmacists identified 77 drug-related problems in 30 patients.

Considering the advancing informatisation of healthcare in Poland, pharmacists can also play a role in assisting patients in setting up Internet Patient Accounts (IPAs). These accounts consolidate comprehensive medical records, including e-referrals and e-prescriptions, making them accessible to selected medical professionals. This access is particularly valuable for pharmacists, enabling them to verify information on administered medications.

## 6. Conclusions

Pharmacotherapy stands as a fundamental component of patient treatment, underscoring the critical role of pharmacists’ knowledge and experience in maximising treatment outcomes and ensuring the highest possible quality of life for patients.

The implications of drug-related problems and non-adherence are profound, impacting health, economy and society. Pharmaceutical services centred on prevention, education, uninterrupted treatment provision, and supporting patients in resolving treatment issues can significantly alleviate the strain on healthcare system. Consequently, pharmaceutical care should emerge as a cornerstone of primary healthcare and a key objective within national health policies.

Efficient provision of pharmaceutical services necessitates the specification of requirements for pharmacies concerning premises, equipment, and staff. It is equally vital to establish essential qualifications for pharmacists offering specific services and ensure comprehensive training in these areas. The development of healthcare funding principles, including provisions for financing from public funds, is of paramount importance in this context.
